# Phytochemical screening, antioxidant and anti-Parkinson activities of *Berula erecta*: A novel medicinal plant

**DOI:** 10.1371/journal.pone.0305751

**Published:** 2024-11-15

**Authors:** Asif Khan, Fizan Ullah, Huda Mohammed Alkreathy, Mushtaq Ahmed, Rahmat Ali Khan

**Affiliations:** 1 Department of Botany, University of Science & Technology Bannu, Bannu, KPK, Pakistan; 2 Faculty of Medicine, Department of Pharmacology, King Abdulaziz University, Jeddah, Saudi Arabia; 3 Department of Biotechnology, University of Science & Technology Bannu, Bannu, KPK, Pakistan; The University of Texas at El Paso, UNITED STATES OF AMERICA

## Abstract

*Berula erecta* L. is traditionally used for the treatment of various human ailments. The present project was arranged to study the antioxidant and anti-Parkinson efficacy of *B*. *erecta* extracts against rotenone-induced Parkinson diseases in rats. Fine powder of the plant was extracted with methanol and then fractionated through various solvents with increasing order of polarity. Phytochemical screenings were done using standard protocols and High-performance liquid chromatography (HPLC) while *in-vitro* antioxidant activities of plant fractions were evaluated using different free radicals. *In-vivo* anti-Parkinson and oxidative dysfunction experiments were conducted in rats. Results revealed that various fractions possessed flavonoids, alkaloids, terpenoids saponins, tannin, anthraquinon, and phlobatanine, while terpeniods and alkaloids were absent in aqueous fraction. Chromatographic analysis of methanol fraction showed the presence of various bioactive compounds viz., vitexin, orientin, rutin, catechin and myricetin. *In-vitro* antioxidant activities of various fractions of *Berula erecta* (*B*.*erecta*) showed that methanol fraction has remarkable scavenging efficacy of 2,2-Diphenyl-1-picrylhydrazyl (DPPH), beta carotene, and superoxide free radicals followed by chloroform fraction. Free radicals produced by 2,2’-azino-bis (3-ethylbenzothiazoline-6-sulfonic acid (ABTS), Hydrogen peroxide (H_2_O_2_), and hydroxyl free radicals were considerably scavenged by methanol fraction followed by ethyl acetate fractions. *In-vivo* study of animal model showed that methanol fraction has significant recovery effects at behavioural, physiological and biochemical level against rotenone induced Parkinson disease. *B*.*erecta* has significantly improved rotenone-induced motor and nonmotor deficits (depression and cognitive impairments), increased antioxidant enzyme activity, and reduced neurotransmitter changes. It has been concluded from the present data that *B*.*erecta* enhances neurotransmitter levels by alleviating oxidative stress and antioxidant enzyme activity, hence improving motor activity, cognitive functioning, and decreasing depressed behavior. These data suggest that *B*. *erecta* may be a promising medicinal agent for reducing the risk and progression of Parkinson’s disease.

## 1. Introduction

Parkinson’s disease (PD) is a clinical disorder that may be diagnosed. It has a wide range of etiologies and clinical symptoms [1]. The incidence of Parkinson’s disease, a neurological disorder, is rapidly increasing globally, barring an infectious cause [2]. Parkinson’s disease (PD) is growing more and more common in the elderly people. its symptoms include memory loss, mobility issues, and sleep disturbances. Parkinson’s disease (PD) is a complex neurological disorder characterized by the substantia nigra pars compacta (SNpc) losing dopaminergic neurons, which results in a dopamine shortage [3] and unintentional movements. Neurodegenerative illnesses, including Parkinson’s disease (PD), are also mostly caused by mitochondrial malfunction [4].

Although its etiology is still largely understood, alterations in proteostasis, oxidative stress, and neuroinflammation are generally recognized as significant components contributing to the pathophysiology of PD [5,6]. Drugs combining levodopa and carbidopa, such as Sinemet, Paracopa, Rytary, and Duopa, are commonly used as approved therapies for PD motor symptoms [7,8]. However, long-term use of these medications can result in toxicity, depression, ulcers, and hypertension [9,10].

As a result of side effects of allopathic drugs in the current world, having a strong supplemental herbal therapeutic ingredient is essential [11,12]. Sometimes, specific genetic changes can result in the inheritance of Parkinson’s disease (PD) [13]. It has been determined that the SNCA, LRRK2, and PARK2 genes increase the risk of Parkinsonism [14,15]. Exposure to several chemicals, such as pesticides and herbicides, has been associated with an increased risk of PD [16]. The risk of developing PD may be increased by head injuries, toxins, free radicals and other environmental factors [17,18].

When the body’s free radicals and antioxidant levels are unbalanced, oxidative stress occurs, which can damage neurons in the brain and cause Parkinson’s disease. The development of Parkinson’s disease may be affected by mental illness [19,20]. Mitochondria are energy-producing structures in cells, and dysfunction of these structures may contribute to the evolution of Parkinson’s disease [21–23].

Medicinal plant plays a crucial role in the alleviation of Parkinson diseases. Auddy *et al*., [24], reported that ayurvedic doctors in India recommended numbers of medicinal herbs to treat neurodegenerative conditions like Alzheimer’s, Parkinson’s, memory damage, nerve degeneration, and other neuronal disorders. *Mucuna pruriens* have anti-Parkinson and neuroprotective properties in animal models of Parkinson’s disease [25]. Bhangale *et al*., [26] investigated the neuroprotective effects of *Ficus religiosa* (L.) leaf pet ether extract in Parkinson disease caused by 3-nitropropionic acid. Similarly, *Tribulus terrestris* [27], *Myrica esulenta* [28], *Chromolaena odorata* (L) [29], *Sterculia guttata* [30], *Acorus calamus* Linn [31], *Datura metel* [32], and *Momordica dioica* [33] were investigated for the treatment of Parkinsonian disorders.

*B*. *erecta*, is sometimes known as smaller water-parsnip, cut leaf water-parsnip, or narrow-leaved water-parsnip, is a member of the carrot family distributed in Europe, Asia, Australia, and North America. *B*. *erecta* extracts was not testified with their therapeutic practice against neurodegenerative ailments. Consequently, the current study was arranged to investigate phytochemical qualitative analysis of its chemical constituents, antioxidant activity and anti-Parkinson’s activity of *B*. *erecta* against rotenone induced rat model through behavioral and biochemical analysis.

## 2. Materials and methods

### 2.1 Plant collection

The fully matured plant of *B*. *erecta* was collected from District Bannu after its proper scientific identification and its voucher specimens with Accession No. 129-Bot was placed at herbarium in the Department of Botany UST Bannu. Running tap water was used to wash the *B*. *erecta* in order to clean from dust. *B*. *erecta* was shed dried for three weeks before being processed into a fine powder.

### 2.2 Extraction and fractionation

For one-week, fine plant powder was soaked in an 80% methanol solution before being filtered through Whatman 45 filter paper. The filtrate was evaporated separately using a rotary evaporator until crude extracts are obtained from the filtrate.

The crud extract was first diluted in distilled water to create an aqueous extract, which was used to prepare different fractions on the basis of polarity. Different solvents, such as n-hexane, ethyl acetate, chloroform, and butanol, were used in the solvent-solvent partition technique to separate the fractions. Different fractions were collected separately and evaporated using a rotary vacuum evaporator. The dried fractions were stored at 4°C for further investigations.

### 2.3 Phytochemical analysis

Various biochemical analyses protocol was used to assess the secondary metabolites present in *B*. *erecta* various fractions. Standard procedures were used for each of the chemical tests for the presence of tannins, saponins, flavonoids, terpenoids, alkaloids, phlobatannins, cardiac glycosides, couramins, and anthraquinone.

### 2.4 Total phenolic contents estimation

The Atala *et al*.,[34] methodology was to determine phenolic constituents. Samples from plant extract were combined with 10 ml of the folin-Ciocalteau reagent. The mixture was incubated for 10 min followed by the addition of 0.115 mg/ml Na_2_CO_3_. Ascorbic acid was used as a standard. OD was checked at 765 nm to measure the absorbance spectrum. Per g of dried material, the amount of total phenolic was calculated as mg of gallic acid equivalents (GAE).

### 2.5 Test to determine the presence of total flavonoids

Pompilio *et al*., [35] technique was used to determine the total constituents of flavonoids. According to this procedure 0.25 ml of each fraction and 15–250 g/ml rutin concentrations were used. A mixture of each fraction and rutin were mixed with 1.5 ml of deionized water and 5% NH_4_NO_3_ and incubated for 6 min. After incubation 0.2 ml AlCl_3_ (w/v), 1.0 molar sodium hydroxide (NaOH) were added and incubated for 5 min. OD was calculated at 510 nm. The dried fraction of rutin equivalent mg/g extract was used to compute the ratio of flavonoids.

To ensure the correctness of the results, the experiment was repeated three times for each sample.

### 2.6 Chromatographic analysis

#### 2.6.1 Thin layer chromatography of methanol fraction

70 mg of methanol fraction of *B*. *erecta* was mixed with HPLC grade, 1ml methanol. TLC plates coated with silica gel dimension (20 × 20 cm) were pointed 1 cm from each side and heated for forty min at 110° to activate silica gel. Myricetin, catechin, isoquercitin, vitexin, hyperside, rutin, orientin, luteolin and quercetin were used as standard compounds. Each standard compounds and methanol fraction of plant extract was pointed through small glass capillary tube jet on the upper side of the silica coated TLC plate and placed inside the tank composed of CH_3_COOH, C_4_H_8_OH and H_2_O (1׃4׃5) solution which was acting as mobile phase and kept inside until the moment of solution was completed. The plate was removed outside the tank, dried and sprayed with spray for detection of flavonoids constituent under UV The values of retention factor was determined with standard formula.

#### 2.6.2 High Performance Liquid Chromatography (HPLC) of fractions

70 mg of *B*. *erecta* methanol fraction was extracted with 6 ml HCl (25%) and 20 ml methanol. A twice diluted sample was injected inside the HPLC column 20RBAX ECLIPSE, XDB-C18, (5 μm; 4.6×150 mm, Agilent USA) with UV-VIS Spectra-Focus detector. Two solvents TCA (0.05%) and TFA (in 85% ACN) were used for the moment of sample having 1 ml/min flow rate. Various standard compounds having 0.02–0.5 μg weight including vitexin, myricetin, catechin, hyperside, orientin, and rutin were used for comparison in triplicate.

### 2.7 *In-vitro* antioxidant activities

#### 2.7.1 Evaluation of plant extracts for DPPH radical scavenging activity

Various plant fractions were used to determine the DPPH’s scavenging capacity of each fraction [36]. A stock solution of reagents was prepared by dissolving 0.006 mg of DPPH in 100 ml of methanol. 0.2 ml various plant fractions and ascorbic acid was mixed 2.8 ml DPPH solution and incubated for 30 min. Using a spectrophotometer, the absorbance spectrum was recorded and calculated at 517 nm. According to the formula below, the percentage of DPPH inhibition was calculated as follows:

% of DPPH inhibition = [(Absorbance of DPPH-Absorbance of sample) / (Absorbance of DPPH)]. ×100

#### 2.7.2 ABTS cation radical assay

The scavenging of ABTS free cation radicals were carried out by making a minor modification to the protocol described by Brahmi *et al*., [37]. 7mM of ABTS reagent was mixed with 2.45 mM potassium per sulphate reagent. The combination was maintained in the dark for 8 hrs. After incubation 50% methanol solution was used to dilute the sample, yielding absorbance 0.900 (0.02) at 745 nm. Each fraction was mixed with 3.0 ml of the diluted reagent. The absorbance was up to 6 min using the formula when using the percent activity of different fractions;

Scavenging impact (%) = (Control Ab-Sample Ab) / (Control Ab) × 100

#### 2.7.3 Superoxide radical scavenging activity

In this procedure each fraction of *Berula erecta* was combined with nicotinamide adenine dinucleotide reduced (NADH) solution and 0.5 ml of Nitro Blue Trizol. PMS was added, and incubated at 25° for 15 min. Calculations of the absorbance spectrum was carried out at 530 nm [38].

Superoxide Scavenged% = [Absorbance of Control—Absorbance of Sample]/Absorbance of Control x 100

#### 2.7.4 Assay for detection of H_2_O_2_ radicals reducing effect

Ascorbic acid was used as the standard, and different concentrations of each fraction with 0.4 ml H_2_O_2_ (50 mM phosphate buffer, pH 7.4) was used to test for the reduction of the H_2_O_2_ free radical. Phosphate buffer was used as a blank to measure the absorbance of mixtures at 230 nm [39]. Experiment was repeated in replicates to determine the hydrogen per oxide scavenging ability.

Hydrogen peroxide scavenging percentage = [Control absorbance—sample absorbance] /Control absorbance] *100

#### 2.7.5 Determination of total antioxidant activity

The standard method was used to assess the antioxidant’s potency of each fraction [40]. 4 mM ammonium molybdate, 28 mM sodium phosphate and 0.6 M sulfuric acid was mixed to form the reagent solution. From this mixture, 0.1 ml of the reagent solution and 0.1ml of the aliquot solution was combined. The mixture was placed in test tubes lined with silver foil and incubated for 90 min at 90° in a water bath. The optical density spectrum of various fractions was compared with blank spectrum using ascorbic acid as a reference compound at 765 nm. The following formula was used to calculate the compound’s antioxidant capacity:

Total antioxidant effect (%) = [(Control absorbance-sample absorbance) / (Control absorbance)] 100.

#### 2.7.6 Scavenging of hydroxyl radical activity

Standard technique of Gutteridge and Halliwell [41] was used to assess the activity of hydroxyl radical scavengers. 200 ml of sodium or potassium phosphate buffered water (pH 7.4), 100 ml of 300 mM ascorbate to catalyze the process, 500 ml of 2-deoxyribose (2.8 mM), 100 ml of already mixed ferric chloride (100 mM), and 100 ml of the combination were incubated at 37°C for 1h. 1 ml of an aqueous solution containing 2.8% (w/v) TCA, NaOH, and TBA was heated for 15 min. After cooling, the OD was measured at 532 nm, and the percent inhibition was used to determine the formula below:

Hydroxyl radical scavenging (%) = [(Control Absorbance-Sample absorbance) / (Control absorbance)] ×100.

#### 2.7.7 Bleaching assay of β-Carotene

The experiment conducted using a slightly modified version of Tuyen *et al*., [42]. 10 ml of chloroform and 2 mg of beta-carotene were mixed in the initial phase. Then 200 mg of Tween 80 and 20 mg of linoleic acid was mixed and thoroughly shaked. In order to make the beta-carotene linoleate suspension, the chloroform was removed using 50 ml of distilled water, nitrogen, and repeatedly shaking. When an aliquot of extract sample was dissolved in 250 ml of emulsion and incubated for 45° for 2 hrs. The absorbance of each sample was calculated at 470 nm. Ascorbic acid was used as a standard. The % activity of bata-carotene bleaching was calculated by the following equation:

% bleaching of β-Carotene = [(Control Absorbance-Sample absorbance) / (Control absorbance)] ×100.

### 2.8 *In-vivo* anti-parkinson animal model

Adult male 30 Wistar rats were used for *in-vivo* anti-parkinson activities. Rats were bought from National Institute of Health (NIH) Islamabad. The work was started in compliance with the Faculty of Biological Sciences’ ethics committee policies at the University of Science and Technology Bannu.

## 2.8.1 Experimental design

Standard protocols of Parkinson disease induction were used for animal treatment. 30 male adult Wister rat were randomly divided into 5 groups. The rats of control group received 0.5% N/saline p.o. every day. For three weeks, the disease control group received rotenone in 1% DMSO (5 mg/kg per body weight s.c.) after every two days. For 21 days, one hr prior to the administration of rotenone, various groups of tests were administered orally with *B*.*erecta* methanol extract (200 and 400 mg/kg b.w.) after 24 hrs. The standard control group animals also received, one hr before rotenone, oral administration of a mixture of levodopa-carbidopa (200 mg/kg b.w.). After 21 days period, all animals weights were recorded. Throughout the experiment, all animals were treated humanely according to the international criteria approved by ethical committee for the Care and Use of Laboratory Animals. The animals were sacrificed by an overdose of xylazine and ketamine anesthesia at day 22 by cervical dislocation. Brain was removed, cleaned with phosphate buffer and kept for biochemical at -20° in freezer. Serum samples were collected through heart puncture. The collected serum was processed for various biochemical tests.

## 2.8.2 Evaluation parameters

*2*.*8*.*2*.*1*. *Body weight and food intake*. The rats’ body weights were recorded before the trial began and then every week before the behavioral assessments. The amount of food consumed each day was also recorded.

*2*.*8*.*2*.*2*. *Open field test*. For the time being, rats were assessed in the open field. The examination was carried out in a box (10 by 10 cm in size, with walls 40 cm high). It was processed for three minutes and evaluated for latency to move and square number crossing.

*2*.*8*.*2*.*3*. *Catalepsy test*. To measure catalepsy, the bar test was used. A horizontal bar 9 cm tall and parallel to the floor was used in this test. The rats have their front paws in a semi-rearing position and were perched on the bar. A timer was used to time how long the first paw is taken off the bar. The time limit was set at three minutes.

*2*.*8*.*2*.*4*. *Walk-on-Beam test*. The 100 cm 2 box was placed, and the 100 cm beam was installed up from the ground. On the beam, the rat had been set and was permitted to move freely. Successfully across the rat, and an interval was obtained.

*2*.*8*.*2*.*5*. *Evaluation through rotarod*. We tested the rat’s ability to coordinate its muscles using the rotarod equipment. All rat groups latency to fall was monitored as a performance indicator throughout for 2 min rotation of the rod.

*2*.*8*.*2*.*6*. *The footprint screening*. The fore foot and back foot were coated with harmless red and green colors in order to assess the foot print activity. A 10 cm wide by 100 cm long runway was built. It was permissible for the rat to take steps on it. The white paper was spread out on the runway for each rat. Measurements were made of the step’s length, the bases widths and the claws gaps. Centimeters were used for all measurements.

*2*.*8*.*2*.*7*. *Social interaction performance*. A videotape was used to assess the social attraction. Three parameters were examined: (i) active interaction evaluation, (ii) inactive interaction assessment, and (iii) numbers of interactions. A common instrument for analyzing social interaction was the social connection box, which gave rats a regulated environment in which they could interact. It assumed the form of a transparent, rectangle box containing two sections divided by small apertures that permit rodents to communicate across the sides.

*2*.*8*.*2*.*8*. *Intake of sucrose solution*. The rats home cage was utilized to assess sucrose consumption activities. This investigation was carried out on an every day. Two like graded containers were kept in the cage. A 1% w/v sucrose solution was utilized in one bottle, and regular water was used in the other. To eliminate the possibility of confusing effects, the bottles were exchanged daily. The following formula was used to calculate data:

% Sucrose utilization = (Utilization of sucrose / Utilization of normal water) × 100

*2*.*8*.*2*.*9*. *Hidden platform finding in water*. In this study, rats have to find a platform in a pool that exists underneath water. A flattened-top cylindrical of 5 cm radius was positioned 3 cm underneath the water. To disguise the platform, milk was put in the water. The target quadrant was chosen. For the purpose of this experiment, the Water tub had been separated by four sections. Rats from each quadrant were placed in the tank for a 2 min training session. There was a 15 min gap between the two studies. Following a workout session for 60 min, memory was evaluated.

### 2.9 Oxidative stress

#### 2.9.1 Detection of lipid peroxidation

Using the standard method of TBARS experiment was carried out to determine the *B*. *erecta* extract reduction of LPO (lipid peroxidation). The homogenized rat brain content was centrifuged at 4000 x g for 10 min. Malondialdehyde (MDA) level was measured in the supernatant while the particle was discarded. 100 μl of low-speed supernatant were incubated for 60 min at 37°. After the initial period of incubation, acetate buffer, 8.1% SDS, and TBA were pipetted, and a subsequent incubation was carried out for 60 min at 100°. The formation of a bright pink color is a good indicator of how TBA reacts to MDA. Using ice plates to lower the temperature, measurements were collected spectrophotometrically at 532 nm [43].

#### 2.9.2 Detection of Glutathione (GSH) content

The method was used to calculate glutathione content in rat’s brain tissue and effects of *B*. *erecta* extract [44]. A 600 μL solution of 10% trichloroacetic acid was added to the 150 μL reaction mixture (R1) for incubation. It was then centrifuged at 4° for 10 min at 4000 rpm. To the Ellman’s reagent, we blended 300 μL of supernatant with 600 μL of 0.1 M phosphate buffer. The wavelength of yellow-colored products was determined at 412 nm.

### 2.10 Enzymatic antioxidants

#### 2.10.1 Catalase assay

According to Chance and Maehly, [45] standard approach, a catalase assay was conducted. In several test tubes, the volume of 50 mM phosphate buffer was mixed with 1 ml of brain homogenate. One milliliter of hydrogen peroxide (30 mM) was added to start the reaction. Pipetted phosphate buffer (2.9 ml) was added to hydrogen peroxide (1 ml) to prepare blanks devoid of brain homogenate. After one minute, the optical density decreased in relation to the negative at 240 nm due to hydrogen peroxide’s breakdown. The amount of catalase stated in units is the rate at which 1 M H_2_O_2_ is broken down by the enzyme at a temperature of 25°. Units per milligram of proteins were used to express the particular activity.

#### 2.10.2 Superoxide dismutase (SOD) determination

Based on the method, the assay for SOD involved reducing nitro blue tetrazolium (NBT) forms blue formazan, which is insoluble in water. 1 mL of 50 mM sodium carbonate, 0.4 mL of 24 μM NBT, and 0.2 mL of 0.1 mM EDTA were added to 0.5 mL of brain homogenate. 0.4 mL of 1 mM hydroxylamine hydrochloride was added to the reaction to start it. At 560 nm, the absorbance at zero time was measured, and the absorbance at 25° after 5 min was recorded. In addition, the control was carried out without brain homogenate. The amount of enzyme needed to prevent a 50% reduction in NBT was used to express units of SOD activity. Units of the particular activity were given per milligram of proteins [46].

#### 2.10.3 GPx activity determination

Glutathione peroxidase activity was assayed by the method of Habig *et al*. [47]. The reaction mixture consisted of 1.49 ml phosphate buffer (0.1 M; pH 7.4), 0.1 ml EDTA (1 mM), 0.1 ml sodium azide (1 mM), 0.05 ml glutathione reductase (1 IU/ml), 0.05 ml GSH (1 mM), 0.1 ml NADPH (0.2 mM), 0.01 ml H_2_O_2_ (0.25 mM) and 0.1 ml of homogenate in a total volume of 2 ml. The disappearance of NADPH at 340 nm was recorded at 25°C. Enzyme activity was calculated as nM NADPH oxidized/min/mg protein using molar extinction coefficient of 6.22 × 10^3^ M^-1^cm^-1^.

### 2.11 Statistical analysis

Computerized program of Graph prism pad software was used for *in-vitro* data analyses determination. The significance level of different treatments will be assessed by 0.01 levels of probability and LSD at 0.05% using SPSS software.

## 3. Results

### 3.1 Phytochemical screening

#### 3.1.1 Qualitative analysis

Phytochemical evaluation of an extract of *Berula erecta* exposed the existence of numerous phytoconstituents in the methanol extract flowed in different fractions, i.e., n-hexane, chloroform, ethyl acetate, butanol and aqueous. Different phytochemicals were present which are shown in the Table 1.

**Table 1:** Phytochemical analysis of various fractions of *Berula erecta*.

Fractions	Flavonoids	Alkaloids	Tannins	Terpeniods	Saponins
Butanol	+	+	+	+	+
Methanol	+	+	+	+	+
N-hexane	+	+	+	+	+
Chloroform	+	+	+	+	+
Aqueous	+	-	+	-	+
Ethyl Acetate	+	+	+	+	+

#### 3.1.2 Quantitative analysis of total flavonoids and phenolic compounds

*3*.*1*.*2*.*1 Total phenolic and total flavonoids contents*. Folin–Ciocalteau procedure was used to find the total phenolic compounds in various fractions of *B*. *erecta*. Table 2 revealed maximum concentration of total phenolic contents in methanol fraction (289.56±1.03 mg GAE/g) followed by Butanol (159.45±2.11 mg GAE/g), chloroform (122.34±1.08 mg GAE/g), ethyl acetate (34.21±2.33 mg GAE/g), n-Hexane (19.12±2.34 mg GAE/g) and aqueous (12.45±3.02 mg GAE/g) fractions respectively. Similarly maximum quantities of flavonoids were present in methanol fraction of *B*. *erecta* followed by butanol, chloroform, ethyl acetate, n-Hexane and aqueous fractions.

**Table 2:** Total phenolic and flavonoid components various fractions.

Sample	Total phenolic components as mg gallic acid equivalent (GAE mg/g extract)	Total flavonoids as mg rutin equivalent (mg/g extract)
Methanol	289.56±1.03	35.18±1.08
Butanol	194.45±2.11	24.72±1.35
Chloroform	122.34±1.08	15.32±1.06
Ethyl acetate	34.21±2.33	7.18±0.94
n-Hexane	19.12±2.34	6.25±0.83
Aqueous	12.45±3.02	3.25±0.37

Mean±SE (n = 3).

#### 3.1.3 Chromatographic analysis

*3*.*1*.*3*.*1 Thin layer chromatography of fractions*. Various fractions of *B*. *erecta* were analyzed and their RF values were obtained by comparing standard flavonoid compounds like orientin, myricetin, vitexin, catechin, isovitexin, hyperoside, rutin, quercetin, and luteolin (Table 3). Retention factor (RF) values of various spotted lines were obtained by checking their colors under UV light before 2-aminoethyl diphenyl borinate spraying and after spraying. The different colors revealed that n-Hexane fraction was composed of an unknown compound having dark brown color (0.80 RF), aqueous fraction composed of dark green color catechin (0.74 RF) and light green vitexin (0.65 RF), butanol fraction contain light green vitexin (0.65 RF), dark green catechin (0.74 RF) while chloroform fraction composed of hyperoside (0.70 RF), catechin (0.74 RF) and Myricetin (0.83 RF) and methanol fraction of *B*.*erecta* composed of eight flavonoids including five known compounds viz., orientin (0.44 RF), rutin (0.50 RF), hyperoside (0.70 RF), catechin (0.74 RF) and three unknown compounds having RF values (0.10), (0.22), (0.24) while ethyl acetate extract contain hyperoside (0.70 RF) and orientin (0.44 RF) as presented in Table 4.

**Table 3:** Appearance of standards under UV 365 nm.

Standard	RF Value	Color (Reagent)
Rutin	0.5	Fluorescent yellow
Myricetin	0.83	light orange
Catechin	0.74	dark green
Vitexin	0.65	light green
Orientin	0.48	light green
Hyperoside	0.70	Orange
Isovitexin	0.60	light green
Luteolin	0.57	Fluorescent yellow
Quercetin	0.87	Yellow

**Table 4:** Appearance and RF values of samples under UV 365 nm in various fractions.

Group	Color	RF value	Compound
Aqueous	dark green	0.74	Catechin
	Light green	0.65	Vitexin
Butanol	Light green	0.65	Vitexin
	dark green	0.74	Catechin
Methanol	light green	0.10	Unknown
	dark green	0.22	Unknown
	light green	0.24	Unknown
	light green	0.44	Orientin
	Florescent yellow	0.50	Rutin
	Orange	0.70	Hyperoside
	dark green	0.74	Catechin
	Light orange	0.83	Myricetin
Chloroform	Orange	0.70	Hyperoside
	dark green	0.74	Catechin
	Light orange	0.83	Myricetin
Ethyl acetate	Orange	0.70	Hyperoside
	light green	0.44	Orientin
n-Hexane	Dark brown	0.80	Unknown

Mean±SE (n = 3).

**Fig 1:**
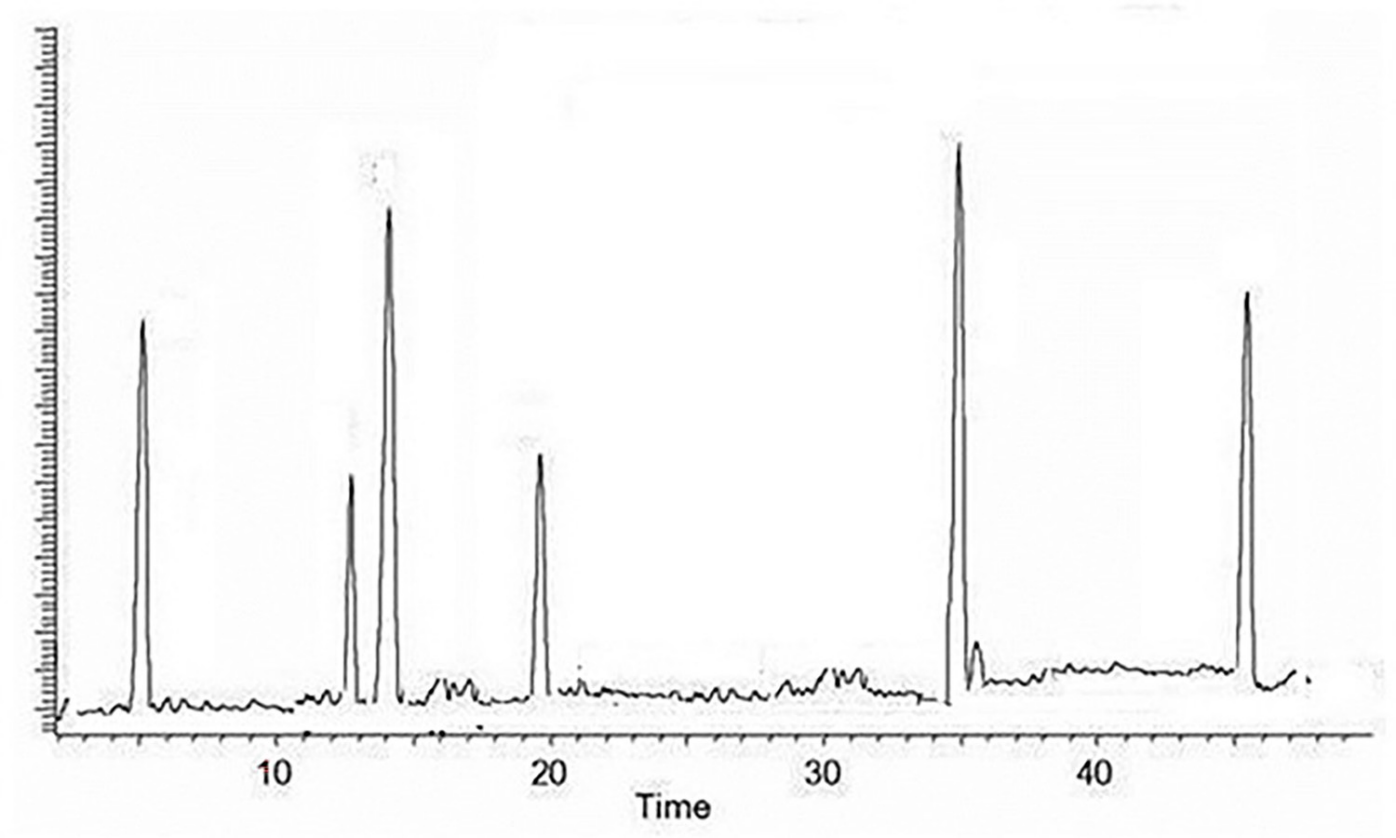
HPLC analysis of methanolic extract of *Berulla erecta* (Column: C18 20RBAX ECLIPSE, XDB-C18, (5 μm; 4.6 × 150 mm, Agilent USA) eluted with mixtures trifluoroacetic acid and acetonitrile).

*3*.*1*.*3*.*2 High performance liquid chromatography (HPLC) of methanol fraction*. In this study, High performance liquid chromatography (HPLC-UV) was selected for the investigation of bioactive flavonoid constituents like rutin, orientin, vitexin, hyperoside, catechin, and myricetin as a standard compound by integration of the peak-areas at 220 nm using an external calibration method as shown in Fig 1. Calibration curves were constructed for each analyte by using a series of standard mixture solutions. Least-squares linear regression was used to determine the calibration parameters for each of the six standards. A summary of the calibration studies for the six analytes was presented in Table 5. The linearity of all calibration curves was determined by calculating the correlation coefficients, which varied from 0.979 for vitexin to 0.9895 for myricetin. The limit of detection (LOD), defined as the lowest detectable concentration of an analyte, was calculated using the formula LOD = (*b*+3*σb*)/*a*, where *a* is the slope of the calibration curve; *b* is the intercept; and *σb* is the standard deviation associated with the intercept. The LOD for the analytes were in the range of 0.85–3.05 ppm. In addition, the limit of quantification (LOQ), defined as the lowest measurable analyte concentration was determined according to the formula LOQ = (*b*+10*σb*)/*a*, where all parameters are as defined for the LOD. The LOQ is reported in Table 6 as the lower limit from the linear range. Chromatogram of methanol fraction revealed the presence of five bioactive compounds orientin, catechin, rutin, myricetin, hyperoside and few peaks of unknown compounds. The relationship between peak area and analyte concentration is expressed as linear regression lines (*y* = *ax* + *b*), where *y* is the peak area measured by UV detector, *x* is the concentration (ppm) of the analytes, and *a* and *b* are the respective slope and intercept of the calibration curve. The correlation coefficient is *r*.

**Table 5:** Calibration of standards.

Compound	Retention time	A	B	R	Linear range (ppm)	LOD (ppm)
Catechin	4.1	625	300	0.983	20–200	3.05
Orientin	12.5	12833	363.3	0.9419	10–170	2.30
Rutin	14.0	12833	133.4	0.9792	4–125	1.60
Hyperoside	19.8	6333	153.3	0.989	6–165	0.85
Myricetin	35.0	5250	110	0.9885	7–250	1.05

Mean±SE (n = 3).

**Table 6:** HPLC-Chromatogram of methanol fraction.

Compound	Retention time	Concentration (μg/mg dry weight)
Catechin	4.1	0.607
Orientin	12.5	0.125
Rutin	14.0	0.698
Hyperoside	19.8	0.135
Myricetin	35.0	0.897

Mean±SE (n = 3).

### 3.2 Antioxidants free radical scavenging assay

**Fig 2:**
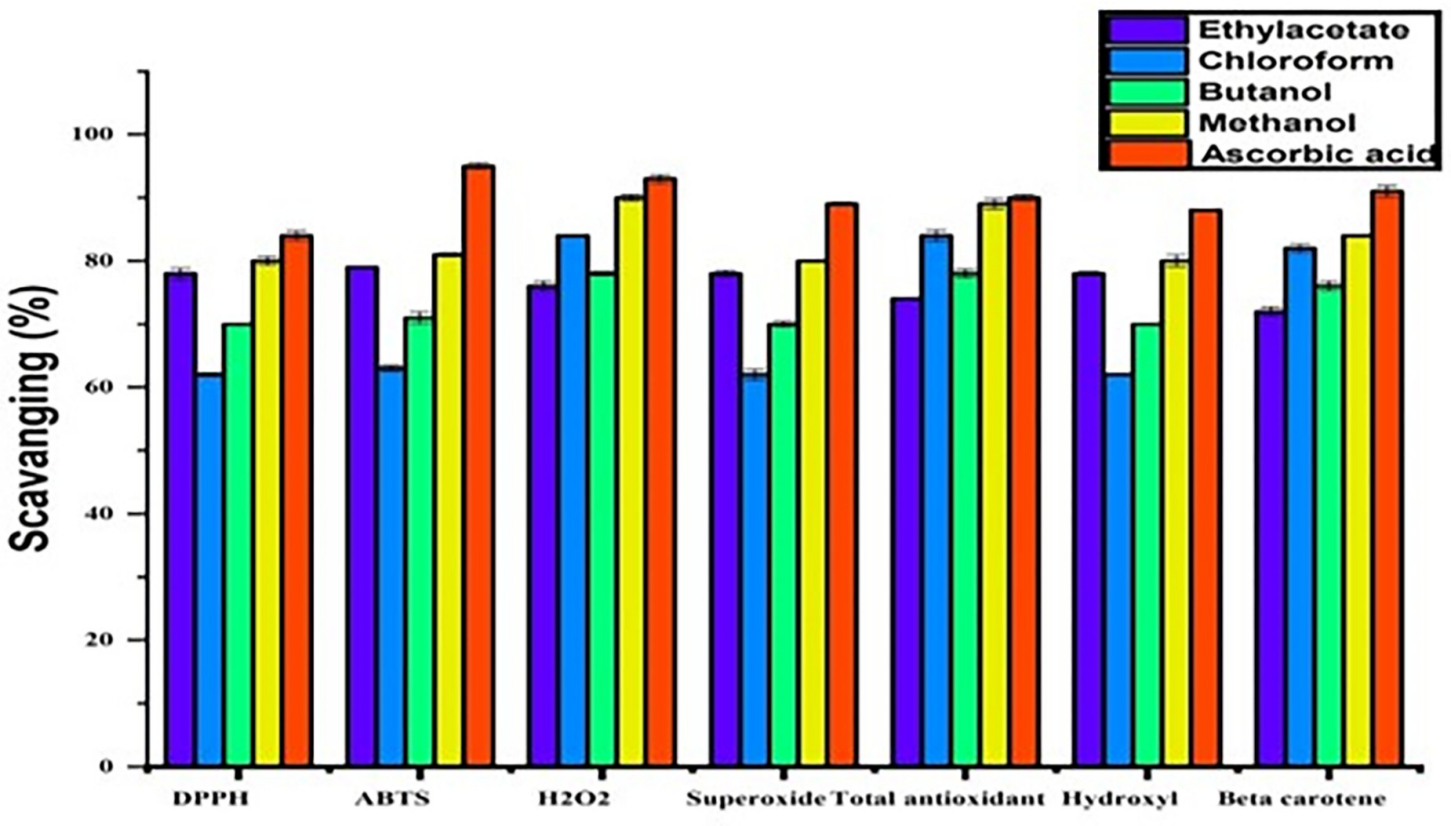
Antioxidants free radical scavenging ability of various fractions of *Berulla erecta*.

*B*.*erecta* anti-free radical scavenging ability was checked using its various fractions as shown in Fig 2. The present finding showed that various fractions are effective in scavenging free radicals. The order of scavenging of DPPH, ABTS, superoxide and hydroxyl radicals were methanol < ethyl acetate < butanol < chloroform while anti-free radical scavenging ability of beta carotene and total antioxidant free radical was reported at the rate of methanol followed by ethyl acetate however ascorbic acid was as a standard antioxidant.

### 3.3 *In-vivo* anti-Parkinson activity

#### 3.3.1 Effects of *B*.*erecta* on body weight

Body weight of experimental rats play a key role in the anti-Parkinson activity of plant extracts. The animals body weight was determined in order to assess their general health. 5 mg/kg body weight rotenone caused non-significant reduction in body weight of rats. *B*. *erecta* 200 mg/kg and 400 mg/kg body weight treatment restored the decrease in the body weight of experimental rats and improved their general health condition dose dependently. Similarly, levodopa-carbidopa showed recovery effects as compare of rotenone induced Parkinson diseased rats as shown in Table 7.

**Table 7:** Effects of *B*. *erecta* on body weight (g) of PD model rats.

Groups	After 7 days	After 14 days	After 21 days
Control	223.41±2.91	233.23±1.93	244.45±3.91
5 mg/kg Rotenone	206.33±2.32	196.50±2.47	184.83±2.83
5 mg/kg Rotenone +200 mg/kg L.carbidopa	218.33±3.45	220.83±4.69	222.33±3.47
5 mg/kg Rotenone +200 mg/kg *B*. *erecta*	210.18±1.93	217.65±2.75	218.03±3.53
5 mg/kg Rotenone +400 mg/kg *B*. *erecta*	219.28±2.76	221.65±2.86	230.63±2.50

Values are represented in Mean ± SEM (*n = 6*).

#### 3.3.2 Effects *B*. *erecta* on food intake

The food intake of all experimental rats (*n = 6*) was measured to investigate the physical general condition of experimental rats. The normal control group rats did not show any significant change. Rotenone induced Parkinson diseased rats showed non-significant reduction in the intake of food which revealed complications in rats. The rats treated with 200 mg/kg and 400 mg/kg body weight *B*. *erecta* methanol extract presented improvement and increased the food intake dose dependently; however, the rats administered with 200 mg/kg body weight L. carbidopa showed normal food intake as were shown in Table 8.

**Table 8:** Effects of *B*. *erecta* on food intake (g) of PD model rats.

Groups	After 7 days	After 14 days	After 21 days
Control	105.71±1.49	106.57±1.97	107.29±3.80
5 mg/kg Rotenone	100.57±3.82	93.40±2.73	85.99±2.46
5 mg/kg Rotenone +200 mg/kg L. carbidopa	104.19±5.61	104.83±2.50	105.43±4.54
5 mg/kg Rotenone +200 mg/kg *B*. *erecta*	102.57±5.07	102.93±4.42	103.23±2.36
5 mg/kg Rotenone +400 mg/kg *B*. *erecta*	104.86±3.56	105.33±3.72	106.24±2.38

Values are represented in Mean ± SEM (*n = 6*).

#### 3.3.3 Rotarod test and latency to cross the beam

Behavioral markers play a crucial role in the assessment of Parkinson activities of plant extracts. To measure muscular strength, a rotarod test was repeatedly used. In comparison to the healthy control group rats, the rotenone induced Parkinson induced diseased control group rats revealed significant reduction (P<0.01) and took less time of falling as compare to normal control group rats. Rats treated with 200 and 400 mg/kg body weight of *B*. *erecta* showed significantly (P<0.01) lengthened time before falling. Similar results were inferred by the treatment of 200 mg/kg body L. carbidopa (Table 9).

**Table 9:** Effects of *B*. *erecta* on motor symptom of PD model rats (Rotarod test).

Groups	Time of falling (S)	Time of falling Crossing the Beam (S)
Control	55.12±1.46++	8.34±0.73++
5 mg/kg b.w. Rotenone	7.45±1.64**	37.23±0.57**
5 mg/kg Rotenone +200 mg/kg L. carbidopa	45.61±1.54++	13.50±0.56++
5 mg/kg Rotenone +200 mg/kg *B*. *erecta*	24.83±1.56++	22.16±0.73++
5 mg/kg Rotenone +400 mg/kg *B*. *erecta*	48.26±2.61++	10.61±0.87++

Values are represented in Mean ± SEM (*n = 6*)

**Significance from the control group at P<0.01 probability levels and

++Significance from the Rotenone group at P<0.01 probability level.

Coordination system plays an important role in Parkinson disease. A beam walking test was to check motor and coordination states. The beam was 100 cm long. The latency to cross the beam and time of falling was significantly (P<0.01) increased in the rats treated with rotenone as compare to non-treated control group rats. 200 mg/kg and 400 mg/kg body weight administration of *B*. *erecta* significantly reduced (P<0.01) the time of falling dose dependently. Similarly, 200 mg/kg body weight L. carbidopa showed significant reduction (P<0.01) of time falling as compare to rotenone induced rats as displayed in Table 9.

#### 3.3.4 Open Field Test (OFT) and cataleptic condition test

An open-field test paradigm was to track locomotors and ambulatory activities in rats (*n = 6*). Data revealed that rats treated with rotenone showed significantly increased (P<0.01) the latency to move while decreased the crossing of number of squares during this study during time period of 2 min. The rats treated with 200 mg/kg and 400 mg/kg body weight *B*. *erecta* methanol extract and similarly 200 mg/kg body weight L. carbidopa significantly decreased (P<0.01) the time of movement and crossing of squares. An inclined plane or bar test was used to diagnose catalepsy. The percentage of cataleptic score measurement revealed that rotenone induced diseased rats showed significant (P<0.01) increase in percent cataleptic score as compare to normal non treated rats. The administration of 200 mg/kg and 400 mg/kg body weight of *B*. *erecta* presented significant improvement (P<0.01) and reduced the score dose dependently. Rats treated with 200 mg/kg body weight L. carbidopa also significant (P<0.01) reversed the cataleptic score (Table 10).

**Table 10:** Effects of *B*. *erecta* on open field test of PD model rats.

Groups	Latency to move (s)	Number of squares to crossed (cm)	% Cataleptic score
Control	1.52±0.08++	157.73±3.94++	0.57++
5 mg/kg Rotenone	9.21±0.16**	50.38±2.98**	5.06**
5 mg/kg Rotenone +200 mg/kg L. carbidopa	2.76± 0.13++	138.55±3.48++	1.25++
5 mg/kg Rotenone +200 mg/kg *B*. *erecta*	6.16±0.07++	137.70±2.50++	2.66++
5 mg/kg Rotenone +400 mg/kg *B*. *erecta*	3.36± 0.13++	148.60±3.38++	1.87++

Values are represented in Mean ± SEM (*n = 6*)

**Significance from the control group at P<0.01 probability levels and

++Significance from the Rotenone group at P<0.01 probability level.

#### 3.3.5 Footprint test

In this investigation, the rat’s footprints were also collected to analyze their stride length and base width patterns of various experimental rats. 5 mg/kg body weight Rotenone induced in rats caused altered stride and base patterns, by noticeably shorter forelimb and hind limb strides in comparison to control animals. The front base width and the hind base width both significantly decreased (P<0.01) and increase paw overlap as well. Rats treated with 200 mg/kg and 400 mg/kg body weight *B*. *erecta* showed marked improvement (P<0.01) and reversed the changed caused by treatment of rotenone in rats. The standard drug 200 mg/kg body weight L. carbidopa showed significant (P<0.01) results as compare to rotenone induced rats (Table 11).

**Table 11:** Effects of *B*. *erecta* on walking by footprint test of PD model rats.

Groups	Stride length(cm) Forelimb	Stride length(cm) Hind limb	Base width(cm) Fore base	Base width(cm) Hind base	Paw overlap (cm)
Control	13.60±0.34++	15.16±0.31++	6.28±0.08++	6.68±0.06++	0.16±0.03++
5 mg/kg Rotenone	3.96±0.26**	5.50±0.21**	3.21±0.06**	3.46±0.04**	1.31±0.03**
5 mg/kg Rotenone +200 mg/kg L. carbidopa	12.80±0.24++	13.11±0.26++	6.11±0.09++	5.83±0.04++	0.38±0.03++
5 mg/kg Rotenone +200 mg/kg *B*. *erecta*	8.53±0.31++	9.60±0.18++	4.96±0.06+	4.06±0.07+	0.71±0.04+
5 mg/kg Rotenone +400 mg/kg *B*. *erecta*	13.50±0.48++	12.61±0.41++	6.01±0.02++	5.35±0.01++	0.36±0.02++

Values are represented in Mean ± SEM (*n = 6*)

**Significance from the control group at P<0.01 probability levels and

++Significance from the Rotenone group at P<0.01 probability level.

#### 3.3.6 Social interaction test

To track depressive-like behavior, a social interaction test was performed. 5 mg/kg body weight administration of rotenone in rats caused significant reduction (P<0.01) in social integrations as compared to non-treated normal control rats. Treatment of 200 mg/kg and 400 mg/kg body weight *B*. *erecta* showed marked improvement in social behavior and significantly increased (P<0.01) the number of interactions, both active and passive. Similarly, 200 mg/kg body weight L. carbidopa markedly (P<0.01) boosted the quantity of interactions, both active and passive, in comparison to animals that had received rotenone injections (Table 12).

**Table 12:** Effects of *B*. *erecta* on PD model rats of social interaction test.

Groups	No of interaction	Active interaction	Passive interaction
Control	17.50±0.619++	23.00±0.447++	17.16±0.37++
5 mg/kg Rotenone	4.33±0.421**	06.66±0.494**	1.50±0.48**
5 mg/kg Rotenone +200 mg/kg L-carbidopa	13.83±0.307++	21.16±0.307++	15.50±0.56++
5 mg/kg Rotenone +200 mg/kg *B*. *erecta*	9.83±0.307++	14.83±0.307++	4.16±0.41++
5 mg/kg Rotenone +400 mg/kg *B*. *erecta*	12.43±0.317++	20.06±0.207++	16.30±0.34++

Values are represented in Mean ± SEM (*n = 6*)

**Significance from the control group at P<0.01 probability levels and

++Significance from the Rotenone group at P<0.01 probability level.

#### 3.3.7 Sucrose consumption test

The inability to enjoy the pleasure that sugar provides was described as anhedonia in rats. Rotenone-treated rats consumed significantly less (P<0.01) sucrose solution than the untreated normal control rats. However, after 24 hrs, 48 hrs, and 72 hrs, rats treated with 200 mg/kg and 400 mg/kg body weight *B*. *erecta* as well as 200 mg/kg body weight consumed significantly more (P<0.01) sucrose solution as compare rotenone treated rats as shown in Table 13.

**Table 13:** Effects of *B*.*erecta* on PD model rats of Depressive-like symptom.

Groups	% Sucrose consumption		
	24 h	48 h	72 h
Control	62.8 ± 0.2++	25.5 ± 0.3++	17.1 ± 0.4++
5 mg/kg Rotenone	14.2 ± 0.1**	57.1 ± 0.5++	52.2 ± 0.5**
5 mg/kg Rotenone +200 mg/kg L.carbidopa	51.4 ± 0.3++	35.7 ± 0.2++	28.5 ± 0.9++
5 mg/kg Rotenone +200 mg/kg *B*. *erecta*	30.0 ±0.9++	31.6 ± 0.8++	48.2 ± 0.7++
5 mg/kg Rotenone +400 mg/kg *B*. *erecta*	56.0 ±1.1++	27.2 ± 0.5++	28.6 ± 0.8++

Values are represented in Mean ± SEM (*n = 6*)

**Significance from the control group at P<0.01 probability levels and

++Significance from the Rotenone group at P<0.01 probability level.

#### 3.3.8 Morris water maze test

The impact of the therapy on memory performance was investigated using the Morris water maze test. Table 14 revealed that rotenone injection considerably increased (P<0.01) escape latency and dramatically decreased (P<0.01) time spent in and crossings over the target quadrant in comparison to the control rats. *B*. *erecta* 200 mg/kg and 400 mg/kg body weight treatment significantly increased (P<0.01) time spent in and crossing over the target quadrant and lowered escape latency as compared to the rotenone group.

**Table 14:** Effects of *B*.*erecta* on Morris water maze test.

Groups	Escape latency (s)	Time spent in target quadrant (s)	Number of Crossing over target quadrant
Control	2.00±0.36++	25.83±0.47++	8.50±0.42++
5 mg/kg Rotenone	10.33±0.42**	6.00±0.57**	2.00±0.25**
5 mg/kg Rotenone +200 mg/kg L. carbidopa	2.33±0.33++	22.33±0.49++	7.83±0.30++
5 mg/kg Rotenone +200 mg/kg *B*. *erecta*	5.50±0.42++	11.00±0.51++	5.50±0.42++
5 mg/kg Rotenone +400 mg/kg *B*. *erecta*	3.03±0.43++	19.83±0.59++	6.89±0.20++

Values are represented in Mean ± SEM (*n = 6*)

**Significance from the control group at P<0.01 probability levels and

++Significance from the Rotenone group at P<0.01 probability level.

#### 3.3.9 Oxidative biomarkers test

Rat brain samples were examined for oxidative stress indicators such as TBARS and GSH levels. Rats treated with 5 mg/kg body weight rotenone caused significant increase (P<0.01) in TBARs contents and reduced the level of GSH. Co-treatment of rats with 200 mg/kg and 400 mg/kg body weight *B*. *erecta* showed significant improvement (P<0.01) by reversing the abnormal changed caused by rotenone in the level of TBARs and GSH as compare to the rotenone-treated group. Similarly, markedly decreased (P<0.01) TBARs levels and increased GSH levels was reported in L. carbidopa treated rats as compared to rats treated with rotenone. Activities of antioxidant enzymes revealed the efficiency of body defense system. Administration of 5 mg/kg body weight rotenone caused significant reduction (P<0.01) in the activities of antioxidant enzyme such as superoxide dismutase (SOD), catalase (CAT), and glutathione peroxidase (GPx). The administration of *B*. *erecta* (200 mg/kg and 400 mg/kg) body weight significantly increased (P<0.01) the activities of SOD, CAT and GPx. Similar observation were recorded by the treatment of 200 mg/kg body weight L. carbidopa (Table 15).

**Table 15:** Effects of *B*. *erecta* on Oxidative markers in PD model rats.

Groups	TBARS (nmol/ml)	GSH (μmol/mg protein)	CAT (U min/mg protein)	GPx (Unit/mg protein)	SOD (U/min/mg protein)
Control	17.7± 0.22++	52.8±0.6++	26.70±0.52++	19.64±0.32++	57.23±0.61++
5 mg/kg Rotenone	42.2± 0.44**	13.5±0.4**	12.01±0.61**	8.26±0.54**	14.44±0.35**
5 mg/kg Rotenone +200 mg/kg L. carbidopa	21.8± 0.27++	43.2±0.1++	24.97±0.57++	17.47±0.47++	47.57±0.27++
5 mg/kg Rotenone +200 mg/kg *B*. *erecta*	32.2± 0.51++	21.1±0.5++	22.23±0.48++	15.05±0.72++	29.18±0.61++
5 mg/kg Rotenone +400 mg/kg *B*. *erecta*	27.7± 0.46++	32.6±0.3++	26.52±0.35++	19.31±0.36++	35.09±0.32++

Values are represented in Mean ± SEM (*n = 6*)

**Significance from the control group at P<0.01 probability levels and

++Significance from the Rotenone group at P<0.01 probability level.

### 3.4 Liver profile test

In our study, the liver profile test was also determined in various treated groups. Administration of 5 mg/kg body weight of rotenone caused significant changes (P<0.01) in the liver profile of rats. Treatment of 200 mg/kg and 400 mg/kg body weight *B*. *erecta* improved the abnormalities in SGPT, serum albumin, total protein, and total bilirubin as rotenone treated rats. Similar effects were reported by rats treated with 200 mg/kg body weight L. carbidopa group rats as presented in Table 16.

**Table 16:** Effects of *B*.*erecta* on liver profile test.

Groups	SGPT(ALT) (U/L)	Serum Albumin (μmol/L)	Total Proteins (g/dL)	T.Bilirubin (mg/dL)
Control	26.25 ± 1.15++	3.15 ± 0.35++	6.66 ± 0.14++	1.41 ± 0.44++
5 mg/kg Rotenone	45.95 ± 1.35**	1.05 ± 0.15**	3.37± 0.15**	6.35 ± 0.51**
5 mg/kg Rotenone+200 mg/kg L. carbidopa	27.90 ± 0.80++	3.10 ± 0.10++	6.29 ± 0.23++	2.03 ± 0.17++
5 mg/kg Rotenone+200 mg/kg *B*. *erecta*	33.35 ± 1.15++	2.15 ± 0.25++	5.82 ± 0.47++	7.67 ± 0.28++
5 mg/kg Rotenone+400 mg/kg *B*. *erecta*	30.65 ± 1.55++	3.00 ± 0.10++	6.09 ± 0.51++	4.51 ± 0.19++

Values are represented in Mean ± SEM (*n = 6*)

**Significance from the control group at P<0.01 probability levels and

++Significance from the Rotenone group at P<0.01 probability level.

### 3.5 Kidney profile test

Kidney function and profile play a key role in the health of any individual. Administration of 5 mg/kg body weight rotenone caused significant elevation (P<0.01) in serum creatinine and blood urea level. Co-treatment of 200 mg/kg and 400 mg/kg body weight *B*. *erecta* significantly reversed (P<0.01) the serum creatinine and blood urea dose dependently. Similar reports were obtained from the treatment of rats with 200 mg/kg body weight L. carbidopa (Table 17).

**Table 17:** Effects of *B*.*erecta* on kidney profile test.

Groups	Serum Creatinine (mg/dL)	Blood Urea (mg/dL)
Control	0.5±0.5++	19.5±0.3++
5 mg/kg Rotenone	0.9±0.1**	35.5±0.5**
5 mg/kg Rotenone +200 mg/kg L. carbidopa	0.6±0.1++	20.0±0.5++
5 mg/kg Rotenone +200 mg/kg *B*. *erecta*	0.5±0.5++	16.4±0.7++
5 mg/kg Rotenone +400 mg/kg *B*. *erecta*	0.4±0.3++	18.6±0.2++

Values are represented in Mean ± SEM (*n = 6*)

**Significance from the control group at P<0.01 probability levels and

++Significance from the Rotenone group at P<0.01 probability level.

## 4. Discussion

Due to their efficiency, sustainability, and local accessibility, herbal medicines are increasingly being to treat pathogenic disorders [48]. Due to the drawbacks of the currently available synthetic medications, innovative pharmacological therapeutic agents derived from plants have been discovered [49].

In the current experiment, the phytochemical composition, antioxidant capacity, and anti-Parkinson activity of the medicinal plant *B*. *erecta* were assessed. Qualitative phytochemical analysis of various fractions revealed that alkaloids, tannins, glycosides, saponins, and flavonoids were all present in the plant methanol crude extract. In the crude plant extract of *B*. *erecta*, other phytochemicals including steroids, terpenoids, and saponins were also discovered. Previous research has suggested that plant extracts include a variety of phytochemicals, including steroids, terpenoids, flavonoids, saponins, and alkaloids. These phytochemicals are vital for both the prevention and treatment of infectious diseases, as well as serving as free radical scavengers [40].

Quantitative assessment showed that methanol fraction of *B*. *erecta* plant extract showed maximum quantity of total phenolic and flavonoids contents as compare to other fractions. Similar reports were inferred by other studies [39].

Chromatographic studies such as thin layer chromatography show that various fraction of *B*. *erecta* showed the presence of bioactive flavonoids like orientin, catechin, rutin, myricetin, hyperside by comparing with standard compounds which were confirmed by High performance liquid chromatography. Nour et al., [50] have reported similar findings.

During current studies, the various solvent fractions of *B*. *erecta* exhibited antioxidant activity measured by DPPH, ABTS and H_2_O_2_ scavenging methods. In DPPH, the value of methanol extracts was highest; in ABTS, butanol showed the highest percentage of inhibition, but in H_2_O_2_, ethyl acetate shows the highest percentage of scavenging activity. Previous studies have demonstrated that antioxidant behavior is one of the most frequently identified biological functions of biologically active chemicals that reduce the effects of oxidative stress.

The extract of *B*. *erecta* at 200 mg/kg and 400 mg/kg body weight dose level, recovered the body weight and food intake as compared the disease control group. In the previous study, in comparison to the rats in the rotenone group, rats given both safflower flavonoid extract and Madopar showed significant recovery effects which are in alliance with our finding [51].

The time of falling on rotarod and sucrose consumption is minimum in rotenone treated group as compared to normal control group. The plant *B*. *erecta* shows positive results in rotarod and sucrose consumption tests. The earlier research showed that the rotenone group, period of falling was substantially shorter than control rats. Similar finding has been obtained during the investigation of quercetin pre and post-supplementation against rotenone induced Parkinson disease which has been significantly prolonged the period of falling. The earlier research also showed that rats given rotenone took significantly less sucrose solution than untreated rats. In contrast, groups given quercetin supplements drank greater quantities of sucrose solution over 24 hrs, 48 hrs, and 72 hrs than did the rotenone-only group [52].

Latency to cross beam and cataleptic score is minimum in normal non treated control group rats as compared to disease control rats. Rotenone caused sluggishness in motion due to rigidity of muscle. *B*. *erecta* (200 gm/kg and 400 mg/kg b.w) showed significant recovery effects in rats. Our study findings are consistent with earlier research which showed that rotenone-injected rats showed a significantly higher cataleptic score and latency to walk on beam when compared to control rats. When compared to the group receiving only rotenone injections, the curcumin+rotenone group significantly reduced cataleptic scores and latency to walk on beam [52].

In open field test latency to move rotenone induced Parkinson diseased group showed higher movement than normal control group rats but the number of crossing of squares is low. *B*. *erecta* (200 gm/kg and 400 mg/kg b.w) showed significant improvement as compare to rotenone group rats. Calabrese *et al*., [49] reported similar results during movement in the open field.

5 mg/kg body weight injection caused abnormality in stride length of both forelimb and hindlimb as well as the base width of both fore and hind limp as compared to normal control group. *B*. *erecta* treatment revealed positive effects on rotenone-induced rats footprint test. The outcomes of this education were in agreement with Madiha *et al*. [52] which presented reduced walking outlines and reduced progress distance in rotenone-treated rats.

Antioxidant defense system play crucial role in detoxification of toxic material enter the cell. In the present study rotenone induced Parkinson effect also caused oxidative which ultimately reduced the activity of antioxidant enzymes like SOD and CAT. Various treatments of *B*. *erecta* as well as L.carbidopa revealed significant improvement in enhancement of the antioxidant enzymes activities. Similar findings have been reported by earlier reports [53].

Lipid peroxidation and glutathione contents are very important cellular contents. In the present study rotenone treatment caused significant enhancement in the contents of TBARs and reduction in the level of GSH. Obeso *et al*., [54] obtained similar observation during assessment of tymol against Parkinson disease in PD patient brains homogenate.

Liver is the primary organ inside which highly susptible to any type of toxin. Rotenone induction in rat liver caused ultimately injuries which have increased the serum level of ALT and total bilirubin which revealed that rotenone caused structural and functional damages of hepatic cell which were significant recovered by various doses treatment of *B*. *eretca*. Our outcome has been supported by Liu et al., [55], who reported quercetin significantly protected liver from injuries by recovery liver function biomarkers. The kidney is the energetic organ of the living body which play a crucial part in the maintaining of homeostasis and detoxification and excretion of toxic metabolites from the cell. Rotenone toxic metabolites caused significant alteration in renal serum creatinine and blood urea which revealed that rotenone caused renal toxicity. The serum parameters were significantly reversed by the treatment of *B*. *erecta* as well as L.carbidopa. Amin et al., [56] reported same findings during study of Renoprotective and antioxidant effects of silymarin and propolis on diclofenac sodium-induced renal toxicity in rats.

## 5. Conclusion

The methanol extract showed significant results in improving the various parameters of oxidative dysfunctions and Parkinson disease may be due to the presence of bioactive metabolites. Furthermore, mechanisms of action of these metabolites are encouraged to be investigated.
